# Dynamic peripheral blood microRNA expression landscape during the peri-implantation stage in women with successful pregnancy achieved by single frozen-thawed blastocyst transfer

**DOI:** 10.1093/hropen/hoad034

**Published:** 2023-08-29

**Authors:** Jie Dong, Lu Wang, Yanru Xing, Jun Qian, Xiao He, Jing Wu, Juan Zhou, Li Hai, Jun Wang, Hongya Yang, Jianlei Huang, Xingqing Gou, Ying Ju, Xiyi Wang, Yunan He, Danjie Su, Lingyin Kong, Bo Liang, Xiaohong Wang

**Affiliations:** Department of Gynecology and Obstetrics, Tangdu Hospital, Air Force Medical University, Xi’an, Shaanxi Province, China; Department of Gynecology and Obstetrics, Tangdu Hospital, Air Force Medical University, Xi’an, Shaanxi Province, China; Research Department, Basecare Medical Device Co, Suzhou, China; Research Department, Basecare Medical Device Co, Suzhou, China; Department of Gynecology and Obstetrics, Tangdu Hospital, Air Force Medical University, Xi’an, Shaanxi Province, China; Department of Gynecology and Obstetrics, Tangdu Hospital, Air Force Medical University, Xi’an, Shaanxi Province, China; Department of Gynecology and Obstetrics, Tangdu Hospital, Air Force Medical University, Xi’an, Shaanxi Province, China; Department of Gynecology and Obstetrics, Tangdu Hospital, Air Force Medical University, Xi’an, Shaanxi Province, China; Department of Gynecology and Obstetrics, Tangdu Hospital, Air Force Medical University, Xi’an, Shaanxi Province, China; Department of Gynecology and Obstetrics, Tangdu Hospital, Air Force Medical University, Xi’an, Shaanxi Province, China; Department of Gynecology and Obstetrics, Tangdu Hospital, Air Force Medical University, Xi’an, Shaanxi Province, China; Department of Gynecology and Obstetrics, Tangdu Hospital, Air Force Medical University, Xi’an, Shaanxi Province, China; Department of Gynecology and Obstetrics, Tangdu Hospital, Air Force Medical University, Xi’an, Shaanxi Province, China; Department of Gynecology and Obstetrics, Tangdu Hospital, Air Force Medical University, Xi’an, Shaanxi Province, China; Department of Gynecology and Obstetrics, Tangdu Hospital, Air Force Medical University, Xi’an, Shaanxi Province, China; Department of Gynecology and Obstetrics, Tangdu Hospital, Air Force Medical University, Xi’an, Shaanxi Province, China; Research Department, Basecare Medical Device Co, Suzhou, China; State Key Laboratory of Microbial Metabolism, Joint International Research Laboratory of Metabolic and Developmental Sciences, School of Life Sciences and Biotechnology, Shanghai Jiao Tong University, Shanghai, China; Department of Gynecology and Obstetrics, Tangdu Hospital, Air Force Medical University, Xi’an, Shaanxi Province, China

**Keywords:** embryo implantation, microRNAs, RNA sequence, peripheral blood, gene expression, peri-implantation stage, blastocyst transfer, decidualization, endometrium, enrichment analysis

## Abstract

**STUDY QUESTION:**

What are the dynamic expression features of plasma microRNAs (miRNAs) during the peri-implantation period in women with successful pregnancy via single frozen-thawed blastocyst transfer?

**SUMMARY ANSWER:**

There is a significant change in the plasma miRNA expression profile before and after blastocyst transfer, during the window of implantation.

**WHAT IS KNOWN ALREADY:**

The expression of miRNAs in peripheral blood has indicative functions during the peri-implantation period. Nevertheless, the dynamic expression profile of circulating miRNAs during the peri-implantation stage in women with a successful pregnancy has not been studied.

**STUDY DESIGN, SIZE, DURATION:**

Seventy-six women treated for infertility with a single frozen-thawed blastocyst transfer in a natural cycle were included in this study. Among them, 57 women had implantation success and a live birth, while 19 patients experienced implantation failure. Peripheral blood samples were collected at five different time points throughout the peri-implantation period, including D0 (ovulation day), D3, D5, D7, and D9 in this cycle of embryo transfer. The plasma miRNAs in women with blastocyst transfer were isolated, sequenced, and analyzed.

**PARTICIPANTS/MATERIALS, SETTING, METHODS:**

Peripheral blood samples were collected in EDTA tubes and stored at −80°C until further use. miRNAs were isolated from blood, cDNA libraries were constructed, and the resulting sequences were mapped to the human genome. The plasma miRNAs were initially analyzed in a screening cohort (n = 34) with successful pregnancy. Trajectory analysis, including a global test and pairwise comparisons, was performed to detect dynamic differentially expressed (DE) miRNAs. Fuzzy c-means clustering was conducted for all dynamic DE miRNAs. The correlation between DE miRNAs and clinical characteristics of patients was investigated using a linear mixed model. Target genes of the miRNAs were predicted, and functional annotation analysis was performed. The expression of DE miRNAs was also identified in a validation set consisting of women with successful (n = 23) and unsuccessful (n = 19) pregnancies.

**MAIN RESULTS AND THE ROLE OF CHANCE:**

Following small RNA sequencing, a total of 2656 miRNAs were determined as valid read values. After trajectory analysis, 26 DE miRNAs (false discovery rate < 0.05) were identified by the global test, while pairwise comparisons in addition identified 20 DE miRNAs. A total of seven distinct clusters representing different temporal patterns of miRNA expression were discovered. Nineteen DE miRNAs were further identified to be associated with at least one clinical trait. Endometrium thickness and progesterone level showed a correlation with multiple DE miRNAs (including two of the same miRNAs, hsa-miR-1-3p and hsa-miR-6741-3p). Moreover, the 19 DE miRNAs were predicted to have 403 gene targets, and there were 51 (12.7%) predicted genes likely involved in both decidualization and embryo implantation. Functional annotation for predicted targets of those clinically related DE miRNAs suggested the involvement of vascular endothelial growth factor and Wnt signaling pathways, as well as responses to hormones, immune responses, and cell adhesion-related signaling pathways during the peri-implantation stage.

**LARGE SCALE DATA:**

The raw miRNA sequence data reported in this article have been deposited in the Genome Sequence Archive (GSA-Human: HRA005227) and are publicly accessible at https://ngdc.cncb.ac.cn/gsa-human/browse/HRA005227.

**LIMITATIONS, REASONS FOR CAUTION:**

Although the RNA sequencing results revealed the global dynamic changes of miRNA expression, further experiments examining the clinical significance of the identified DE miRNAs in embryo implantation outcome and the relevant regulatory mechanisms involved are warranted.

**WIDER IMPLICATIONS OF THE FINDINGS:**

Understanding the dynamic landscape of the miRNA transcriptome could shed light on the physiological mechanisms involved from ovulation to the post-implantation stage, as well as identifying biomarkers that characterize stage-related biological process.

**STUDY FUNDING/COMPETING INTEREST(S):**

The study was funded by the Major clinical research project of Tangdu Hospital (2021LCYJ004) and the Discipline Platform Improvement Plan of Tangdu Hospital (2020XKPT003). The funders had no influence on the study design, data collection, and analysis, decision to publish, or preparation of the article. There are no conflicts of interest to declare.

WHAT DOES THIS MEAN FOR PATIENTS? For an early pregnancy to succeed, the embryo must attach to the uterine wall (implant) and continue to develop there. A large number of early pregnancies, however, fail at the stage of implantation. We are interested in testing whether certain biomarkers in blood (plasma) are related to the functional state of the uterus and, consequently, to assess the chance of a successful pregnancy. This study investigated differences between infertile women who became pregnant, and those who did not, after assisted reproduction by measuring levels of small molecules called microRNAs (miRNAs), which are important regulators of gene expression and may be involved in ensuring successful implantation. Other studies have shown that plasma miRNA profiles can be highly predictive of many disease conditions. Plasma levels of miRNAs were measured during the period of embryo implantation in women who received a blastocyst (Day 5–6 embryo) transfer, and we attempted to identify associations between patterns of miRNA levels and embryo implantation. We found that plasma miRNA profiles differ before and after blastocyst transfer, during the critical time period for embryo implantation, and many miRNAs were associated with endometrium thickness (important for implantation) and progesterone (the key pregnancy hormone) levels. These findings, which show that blood levels of many miRNAs undergo large changes during embryo implantation, suggest that the altered miRNAs may be participating in the process of embryo implantation. In the future, it may be possible to use levels of blood miRNAs to assess likely pregnancy outcomes in infertile patients who receive assisted reproduction treatment; this would be a less invasive procedure for the patient.

## Introduction

Embryo implantation is an essential step in the establishment of pregnancy and occurs during a short time frame, termed the ‘window of implantation’ (WOI). Key factors for successful embryo implantation include a viable embryo, appropriate endometrial state, and coordinated embryo–endometrium dialogue (Sharkey and Macklon, 2013). Implantation is a highly complex physiological activity, since the process involves the interactions of a variety of cells (trophoblast, endometrial epithelial, and stromal cells, natural killer cells, and macrophages) at the maternal–fetal interface (Lee *et al.*, 2011; Knöfler and Pollheimer, 2012). It is naturally assumed that the regulatory mechanisms responsible for embryo implantation are intricate and they still remain elusive.

Currently, the regulatory network of embryo implantation has been gradually unraveled based on animal experiments and *in vitro* studies using human samples, showing that successful implantation depends on the organized interaction of various factors including hormones, cytokines, chemokines, growth factors, adhesion molecules, receptors, and lipids (Cha *et al.*, 2012; Zhang *et al.*, 2013). Furthermore, it should be noted that communications among those molecules involved in implantation can be mediated by epigenetic regulatory mechanisms, such as DNA methylation and post-translational modifications, as well as post-transcriptional control of small or long non-coding RNAs (Munro *et al.*, 2010; Rivera and Ross, 2013; Galliano and Pellicer, 2014).

microRNAs (miRNAs) are small non-coding RNAs with 20–30 nucleotides. As one type of epigenetic regulator, miRNAs can regulate various biological processes, usually by inhibiting mRNA translation (Bartel, 2004). It is believed that miRNAs are involved in the process of embryo invasion into the endometrium during WOI (Liang *et al.*, 2017; Paul *et al.*, 2019). Evidence shows that miRNAs are differentially expressed (DE) at different stages of the cycling endometrium, for example in the proliferative versus decidualized endometrium, and the receptive versus pre-receptive endometrium (Paul *et al.*, 2019). Additionally, under pathological conditions, for example recurrent implantation failure, miRNAs exhibit differential expression profiles in diverse samples, such as uterine luminal fluid, endometrial tissues, and peripheral plasma (Revel *et al.*, 2011; Altmäe *et al.*, 2017; Liang *et al.*, 2017; Albayrak *et al.*, 2021; Zeng *et al.*, 2021; Azhari *et al.*, 2022). The findings indicate that miRNAs play a vital role in embryo implantation via local or systemic regulatory mechanisms. However, those data are primarily derived from experiments conducted on *in vitro* specimens, which may not reflect the dynamic characteristics of the implantation process *in vivo*.

Emerging evidence suggests that miRNAs in peripheral blood have indicative functions in early pregnancy, including embryo implantation (Liang *et al.*, 2017; Zhou and Dimitriadis, 2020), and circulating miRNAs are considered to be important biomarkers owing to their informative, stable, and non-invasive features (Liang *et al.*, 2017). Therefore, the dynamic expression profile of blood miRNAs during the WOI could be used to reveal some of the physiological characteristics of embryo implantation. However, there are no studies reporting the dynamic circulating miRNA expression patterns after the human embryo begins to invade the endometrium during the WOI. In this study, by using small RNA sequencing, we first sought to depict the dynamic circulating miRNA profiles at different sequential time points during human embryo implantation in infertile women who underwent frozen-thawed embryo transfer (FET) and achieved a live birth. We also aimed to define the dynamic physiological properties of human embryo implantation before and after implantation based on this plasma miRNA expression landscape.

## Materials and methods

### Study population

This work was approved by the Ethics Committee of Tangdu Hospital, Air Force Medical University (TDLL-202210-15). All participants signed an informed consent form and sampling was performed at the infertility clinic within the Gynecology and Obstetrics Department of the Institution. Eligible subjects were selected from infertile women who underwent a single FET in a natural cycle at our center, between October 2020 and May 2021. The inclusion criteria of the subjects were as follows: maternal age ≤40 years and having a regular menstrual cycle (a cycle length of 21–35 days) with natural ovulation. Infertile couples with tubal factor or male factor were recruited, but patients who had endometriosis, adenomyosis, uterine abnormalities, intrauterine adhesion, hyperprolactinemia, thyroid diseases, and/or autoimmune diseases were excluded from this study. Seventy-six women with a single FET (blastocyst stage) were included. Among them, 57 women had successful implantation and a live birth while for 19 patients, implantation did not occur.

### Sample collection

The patients in the study underwent follicular monitoring via transvaginal ultrasound and sex hormone testing before embryo transfer. The day of ovulation assessed by a gynecologist was based on plasma LH level and release of the dominant follicle was denoted as Day 0 (D0). Generally, the implantation period spans 5–10 days after ovulation (Galliano and Pellicer, 2014); therefore, we collected blood samples at different time points throughout the WOI, including D0, D3, D5, D7, and D9 in this cycle of embryo transfer (Supplementary Fig. S1). For FET, all blastocysts were vitrified on Day 5 or Day 6 (calculated from the day of IVF) according to embryo development, and a single Day-5 or -6 blastocyst was thawed and transferred to the uterus on D5 (calculated from the day of ovulation), before D5 blood sampling. Peripheral blood samples were collected in EDTA tubes at the five time points mentioned above. Briefly, a 5 ml peripheral blood sample was centrifuged at 2000×*g* for 10 min at 4°C, and supernatants were obtained, aliquoted, labeled, and stored at −80°C until analysis.

### Total RNA isolation and small RNA sequencing

Total RNA was isolated from 1 ml of plasma using the miRNeasy Serum/Plasma Advanced Kit (Cat # 217184, Qiagen, Dusseldorf, Germany) according to the manufacturer’s instructions. After extraction, RNA samples were eluted in 20 μl of nuclease-free water. cDNA was made for each sample using TruSeq^®^ Small RNA Library Prep Kit (Cat # RS-200-0024; Illumina, San Diego, CA, USA) based on the poly (A) RT-PCR method. Initially, adapters were attached to the 21- to 23-nt-long miRNAs to allow for PCR amplification and to accurately identify the native miRNA termini during sequencing. Subsequently, cDNA was generated, followed by PCR amplification, during which barcodes (to enable multiplexing) and sequencing index primers were introduced.

### RNA sequencing data processing and mapping

After small RNA sequencing, the raw reads were quality-checked with a customized in-house pipeline (Supplementary Data File S1). After adapter trimming/demultiplexing, reads were mapped to the reference genome (genome build GRCh 38; miRNAs/piRNAs/other non-coding RNAs known sequences). The unmapped reads with >26 bases were removed, followed by collapsing reads to ensure that each sequence only occurs once. The collapsed, filtered, and unmapped reads were used as the valid reads for the next step in the analysis. Since samples were profiled in several batches, pre-processed data were inspected for the presence of batch effects using principal component analysis (PCA).

### Tissue similarity transcription analysis

Tissue-specific miRNA expression patterns have been broadly discussed. In this study, we were interested in testing whether plasma miRNA biomarkers are related to the functional state of the uterus, by analyzing tissues such as endometrium and uterine fluid. Gene expression data series were downloaded from the NCBI Gene Expression Omnibus (GEO) (NIH, Bethesda, MD, USA) for endometrium (GSE86491) and uterine fluid miRNAs (GSE173289). For the endometrium dataset, samples were collected from two time points, namely the proliferative phase and mid-secretory phase (7–9 days after ovulation) of the menstrual cycle. Considering that our samples were collected from the day of ovulation (D0), in the early secretory phase, we only compared the expression of endometrial miRNAs at mid-secretory phase with D5, D7, and D9 plasma miRNAs. For the uterine fluid dataset, samples were collected by flushing with saline on day LH+ 7–9 (similar to the mid-secretory phase). The expression profile was then compared with D5, D7, and D9 plasma miRNAs. Data were normalized, log transformed, and adjusted for batch effects using the comBat function implemented in the R package sva (https://www.r-project.org). PCA was applied to distinguish the miRNA expression profiles of different sample sources.

### Trajectory analyses

To assess the developmental progression of miRNA expression profiles in plasma from D0, D3, D5, D7, to D9, trajectory analysis was employed. Batch effect detection was first conducted to examine potential bias due to different test batches. A total of 170 samples (34 individuals with five time points, as the screening set) were collected to perform RNA sequencing. The tradeSeq method uses the negative binomial generalized additive model and is applicable for bulk time course studies (Van den Berge *et al.*, 2020). For trajectory analysis, two types of statistical testing hypotheses were conducted, namely the global test and pairwise comparison. The global test, which defines whether the average gene expression is significantly changing along time points, is used to detect the dynamic DE miRNAs. In addition, pairwise comparison, which assesses differential expression using the average expression of any two specific time points, is performed to identify DE miRNAs between two stages. In this study, pairwise comparisons were analyzed between any two time points among D0, D3, D5, D7, and D9, resulting in a total of 10 combinations (D0 versus D3, D5, D7, D9; D3 versus D5, D7, D9; D5 versus D7, D9; D7 versus D9). The above strategy was adopted to give a comprehensive inspection of dynamically changing profiles of miRNA expression during the peri-implantation period. Trajectory analyses were conducted using R package tradeSeq (v. 1.4.0) (https://www.r-project.org).

### Fuzzy c-means clustering

Subsequently, all above DE miRNAs were grouped into different clusters using the Mfuzz package in R with the fuzzy c-means algorithm (Kumar and Futschik, 2007) based on their dynamic expression patterns. Clustering for DE miRNAs from the global testing and pairwise comparisons was conducted separately.

### Relevance to clinical traits

To address the relevance of identified DE miRNAs to clinical traits, the linear mixed model was performed. The linear mixed model was employed to account for repeated measures nested within individuals. Baseline information (female age and BMI), basal hormone levels (including FSH and LH), anti-Müllerian hormone (AMH) level, antral follicle count (AFC), endometrial thickness (ET), dynamic estradiol (E2), and progesterone (P) were analyzed. A *P*-value of ≤0.05 was deemed as significant.

### miRNA target prediction

The potential candidate target genes of those clinically relevant DE miRNAs were predicted with TargetScan 7.2, mirTarBase, and the miRDB v6.0 data library. TargetScan predicts biological targets of miRNAs by searching for the presence of conserved 8mer, 7mer, and 6mer sites that match the seed region of each miRNA (Lewis *et al.*, 2005). mirTarBase is based on text mining of functional studies of miRNAs and collects miRNA–target interactions which have been experimentally validated (Huang *et al.*, 2022). miRDB consists of the application of support vector machines in high-throughput sequencing experiments, and the identification of new miRNA target binding and expression downregulation (Chen and Wang, 2020). The regulatory network between the miRNAs and their targets was constructed, which was visualized by utilizing Cytoscape (Shannon *et al.*, 2003).

### Functional enrichment analysis

Gene Ontology (Ashburner *et al.*, 2000), Kyoto Encyclopedia of Genes and Genomes (Kanehisa and Goto, 2000), Reactome gene sets (Fabregat *et al.*, 2018), and WikiPathways (Martens *et al.*, 2021) enrichment analysis of predictive gene signatures were performed using Metascape (Zhou *et al.*, 2019). Enrichment *P*-values were adjusted using Benjamini–Hochberg correction and an adjusted *P*-value of ≤0.05 was used as the significance cutoff.

## Results

### Clinical baseline characteristics

All 170 plasma samples, collected at 5 different time points during peri-implantation, from 34 women who had a live birth were analyzed first (Supplementary Fig. S1), as the screening set. The basic clinical information is presented in Supplementary Table S1. The common clinical characteristics of the subjects were within the normal range, including BMI, basal FSH, basal LH, basal E2, and AFC. After ART, the mean number of oocytes retrieved and embryos obtained among those infertile women was 14.7 and 10.4, respectively. The levels of E2 and P gradually increased in pace with early pregnancy establishment (Supplementary Fig. S2).

### Tissue similarity transcription analysis

Studies have shown that plasma miRNA profiles can be highly predictive of different disease conditions (Mitchell *et al.*, 2008; Backes *et al.*, 2016), with shared miRNA signatures between plasma and tissues. To understand the similarity of miRNA expression between plasma and uterine tissues, tissue similarity comparison on miRNA expression features was performed between plasma and endometrium as well as uterine fluid, based on GEO datasets (GSE86491 and GSE173289, respectively). After batch effect correction, PCA of the samples showed that plasma miRNA expression had high similarity with endometrium but not uterine fluid (Supplementary Fig. S3). The results indicated that the plasma miRNAs may have a close correlation with endometrial miRNA features and may reflect the functional status of endometrium cells.

### Trajectory-related miRNAs

Following small RNA sequencing, mapping results showed that most sequencing counts were composed of miRNAs, accounting for 54.61% of the total reads. Sequencing read counts were mapped and assigned to the reference genome, and a total of 2656 miRNAs with valid read values before filtering were determined.

Initially, data quality assessments were conducted using unsupervised learning methods (Supplementary Fig. S4). PCA and *t*-distributed stochastic neighbor embedding (*t*-SNE) of miRNA sequence data revealed that there were no significant outlier samples (Supplementary Fig. S4C and D).

**Figure 1.**
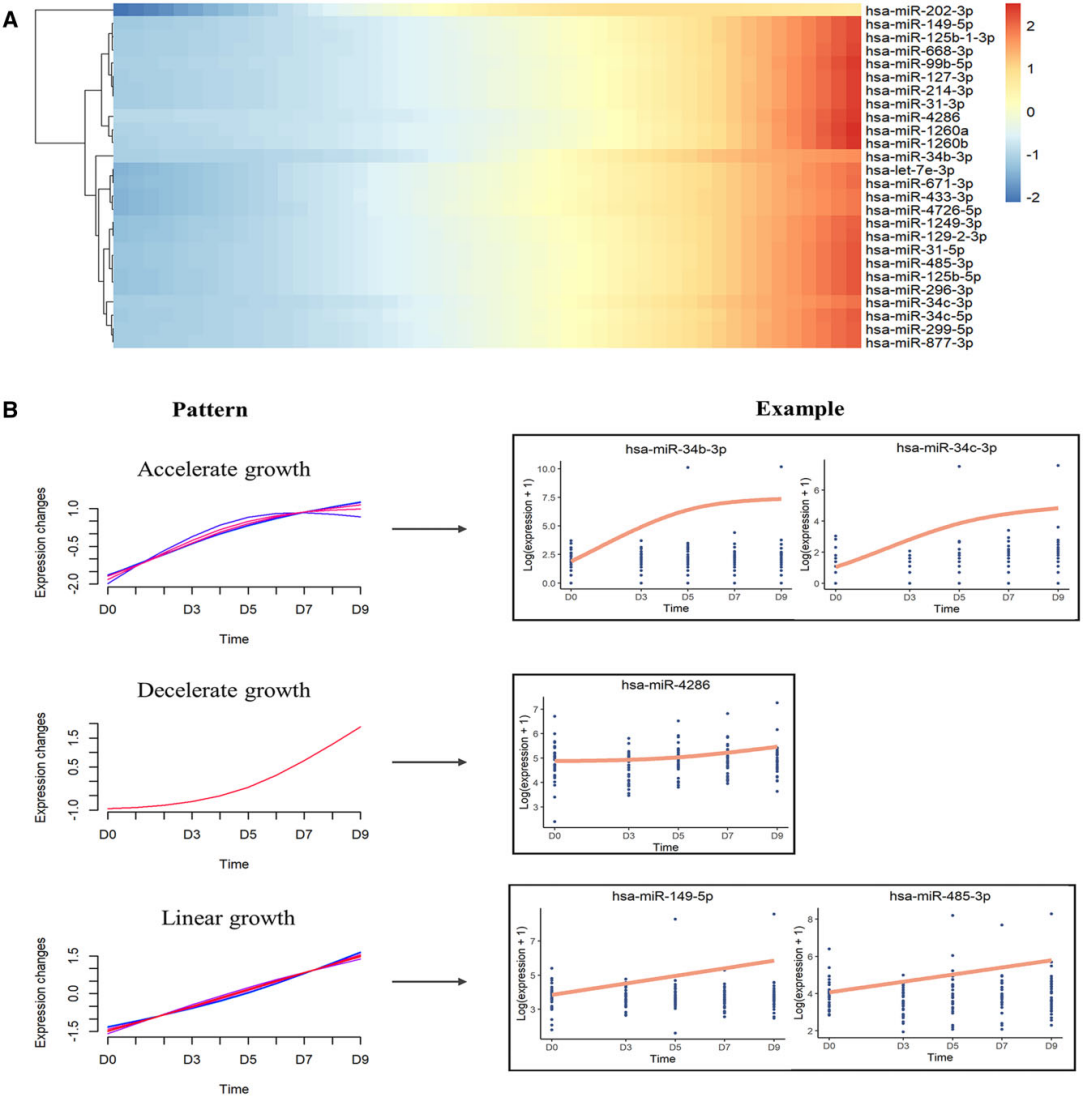
**Overview of dynamic trajectory differential expression profiles.** (**A**) Heatmap plot represents the 26 dynamic differentially expressed (DE) microRNAs (miRNAs) that are associated with the time points. An overall upregulation trend can be identified. (**B**) The left panel shows three distinct temporal patterns of miRNA expression identified by fuzzy c-means clustering. The *x*-axis represents five time stages, while the *y*-axis represents log2-transformed, normalized intensity ratios in each stage. The right panel gives one to two corresponding miRNA expression patterns, as examples. The expression patterns for the remaining dynamic DE miRNAs are provided in Supplementary Fig. S5.

Subsequently, the tradeSeq method was performed to detect dynamic DE miRNAs. The global testing revealed 26 dynamic DE miRNAs, whose expression patterns were changing along time points (Supplementary Table S2). As shown in Fig. 1A, an overall upregulation trend was observed for all dynamic DE miRNAs. Nevertheless, fuzz c-means clustering discovered three distinct upregulation patterns (Fig. 1B, Supplementary Fig. S5, and Supplementary Table S3). The linear growth (LG) pattern included the largest set of DE miRNAs (n = 19), indicating a steady increase trend along time stage. Moreover, we identified two patterns with variable speed of growth. The accelerated growth (AG) pattern consisted of six DE miRNAs while the decelerated growth (DG) pattern contained one DE miRNA (has-miR-4286). Notably, the turning points of AG and DG patterns both occurred around D5, namely the day of blastocyst transfer. Those time-varying patterns may suggest the existence of a miRNA expression transition caused by the occurrence of one key event, namely blastocyst implantation.

**Figure 2.**
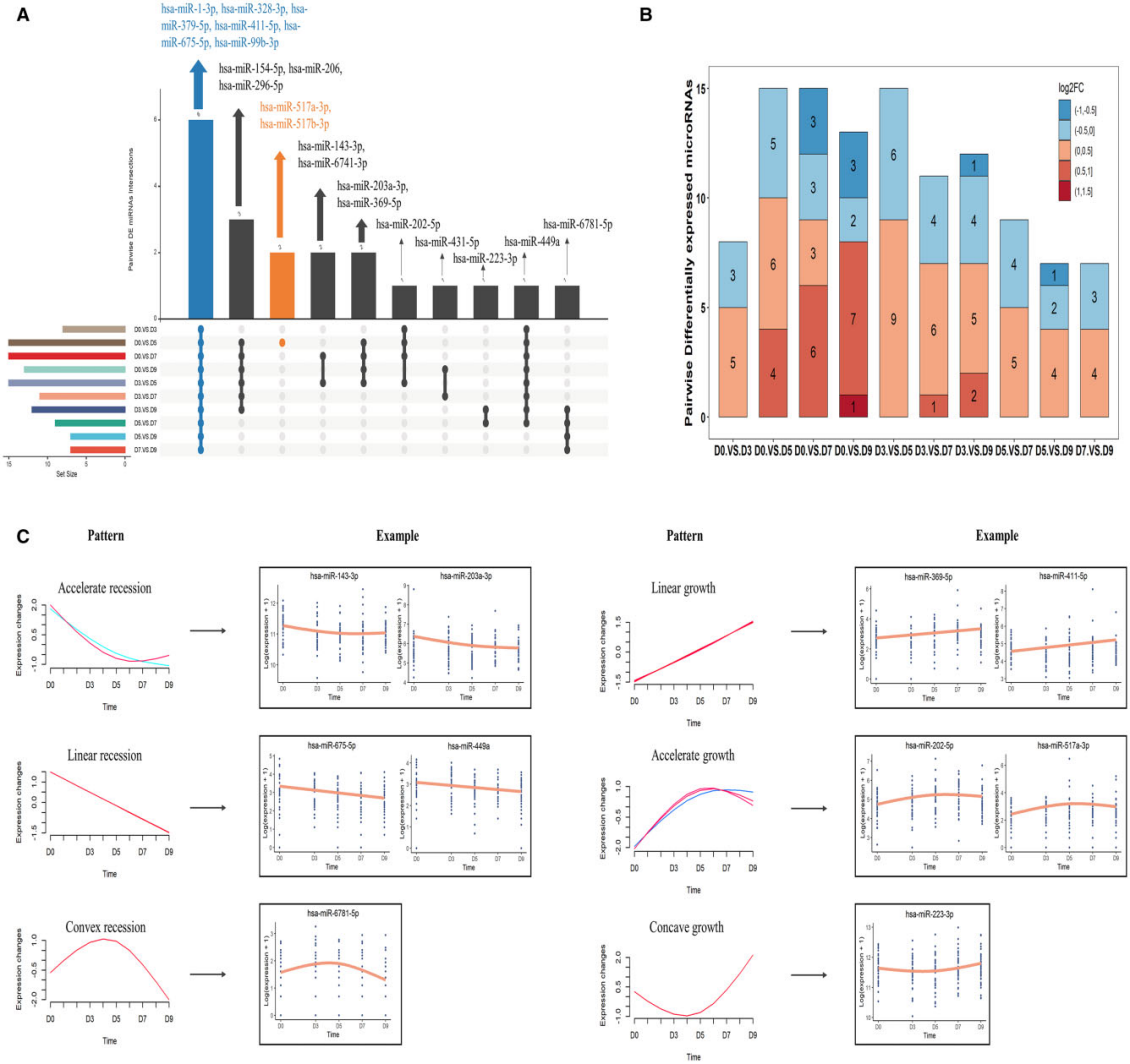
**Overview of pairwise trajectory differential expression profiles.** (**A**) The UpSet plot displays the intersections of 20 pairwise differentially expressed (DE) miRNAs between the sets identified by each pairwise comparison set. The top bar plot shows the intersection number of DE miRNAs. The bottom left bar plots represent the set size of each pairwise comparison set, while the bottom right dot plots represent the intersection situation. For example, the blue dots refer to intersection of all sets and the corresponding bar plot number is 6, meaning 6 DE miRNAs are recognized by all sets. The orange dot and the bar plot number 2 indicate that 2 DE miRNAs are uniquely recognized by the Day 0 (D0) versus D5 comparison set. (**B**) Stacked bar chart shows the quantity changes of DE miRNAs from pairwise comparisons analysis. The red plots represent upregulated DE miRNAs with a false discovery rate (FDR) <0.05 and a log2 fold change >0, while the blue plots represent downregulated DE miRNAs with a log2 fold change <0. (**C**) The left panel shows six distinct temporal patterns of miRNAs expression identified by fuzzy c-means clustering. The *x*-axis represents five time stages, while the *y*-axis represents log2-transformed, normalized intensity ratios in each stage. The right panel gives one to two corresponding miRNAs expression patterns as example. The expression patterns for the remaining pairwise DE miRNAs are provided in Supplementary Fig. S6.

Moreover, we analyzed the differential expression results from pairwise comparisons and found another 20 DE miRNAs between at least two time points (Supplementary Table S4). Figure 2A summarizes the intersections of 20 DE miRNAs between the sets identified by each pairwise comparison. Specifically, six DE miRNAs (hsa-miR-1-3p, hsa-miR-328-3p, hsa-miR-379-5p, hsa-miR-411-5p, hsa-miR-675-5p and hsa-miR-99b-3p) were identified by all sets, while two DE miRNAs (hsa-miR-517a-3p and hsa-miR-517b-3p) were specifically recognized by comparing D0 with D5, corresponding to the day of ovulation and implantation, respectively. As shown in the stacked bar plot (Fig. 2B), the number of upregulated DE miRNAs was greater than downregulated miRNAs in each pair of comparisons. Furthermore, 20 DE miRNAs were grouped into six expression patterns, including accelerated recession (AR), linear recession (LR), convex recession (CR), LG, AG, and concave growth (CG) pattern (Fig. 2C, Supplementary Fig. S6, and Supplementary Table S5). Among them, LG and AG patterns were the same as dynamic DE miRNAs.

Combining the results from both global testing and pairwise comparisons, a total of seven patterns were clustered. Among them, the LR and LG groups showed linear changes along with time, suggesting that their functions may be related to the cycling endometrium. Consistent with the findings from global testing, AR, CR, AG, and CG groups with variable speed along the time stage showed a turning point around D5, suggesting that there is a significant difference in plasma miRNA profile before and after embryo transfer.

### Relevance to clinical traits

**Figure 3.**
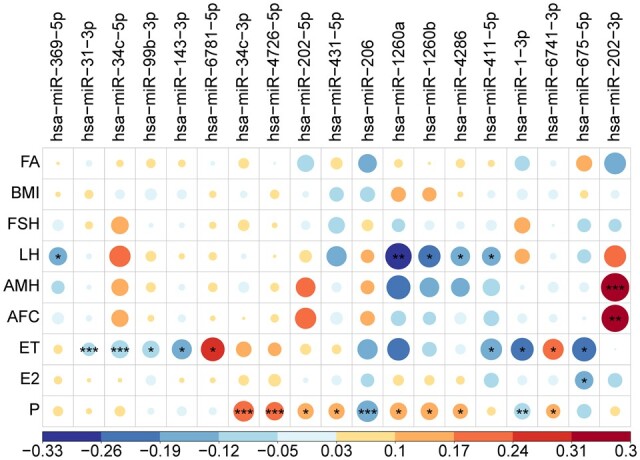
**Correlation matrix plot showing associations between differentially expressed miRNAs and clinical traits using a linear mixed model.** Nineteen differentially expressed (DE) miRNAs with at least one significant trait are shown. The color gradient indicates the direction, i.e. positive (red) and negative (blue), and the strength of the correlation. The significance of *P*-values was labeled as: *0.01 < *P*-value < 0.05, **0.001 < *P*-value < 0.01, ***P-value < 0.001. AFC: antral follicle count; AMH: anti-Müllerian hormone; ET: endometrial thickness; E2: estradiol; FA: female age; P: progesterone.

After identifying DE miRNAs, a linear mixed model was applied to examine the associations between 46 DE miRNAs and 9 clinical traits. As shown in Fig. 3, a total of 19 DE miRNAs exhibited significant associations with at least one clinical parameter. We further clarified those clinically related DE miRNAs based on their expression patterns. As shown in Table 1, 19 DE miRNAs were grouped into six patterns, with pattern LR, LG, and AG including more than one DE miRNA. Turning now to their clinical relevance, it seemed that miRNAs with the same expression pattern showed similar associations with clinical traits. For example, all three DE miRNAs (miR-1-3p, miR-206, and miR-675-5p) in the LR group were negatively associated with ET, E2, or P. In addition, all eight DE miRNAs in the LG group were negatively correlated with LH, ET or positively correlated with P. Interestingly, in contrast to other clinical indices, ET and P showed a correlation with more DE miRNAs (including two of the same miRNAs, hsa-miR-1-3p, and hsa-miR-6741-3p). The correlation results between DE miRNAs and ET, as well as P level, indicated that some miRNAs in the circulation may be engaged in preparation of a receptive endometrium or be influenced by progesterone production.

**Table 1.** Clinical relevance of differentially expressed miRNAs in different clusters.

microRNA	Cluster	Clinical relevance
hsa-miR-1-3p	LR	Negatively correlated with ET, E2, or P
hsa-miR-206	LR	
hsa-miR-675-5p	LR	

hsa-miR-1260a	LG	Negatively correlated with LH and ET or positively correlated with P
hsa-miR-1260b	LG	
hsa-miR-369-5p	LG	
hsa-miR-411-5p	LG	
hsa-miR-31-3p	LG	
hsa-miR-431-5p	LG	
hsa-miR-4726-5p	LG	
hsa-miR-99b-3p	LG	

hsa-miR-4286	DG	Negatively correlated with LH and positively correlated with P

hsa-miR-6781-5p	CR	Positively correlated with ET

hsa-miR-143-3p	AR	Negatively correlated with ET

hsa-miR-202-3p	AG	Positively correlated with AMH, AFC, or P; correlated with ET^a^
hsa-miR-202-5p	AG	
hsa-miR-34c-3p	AG	
hsa-miR-34c-5p	AG	
hsa-miR-6741-3p	AG	

Linear mixed model identifying 19 of 46 differentially expressed (DE) miRNAs that are significantly associated with at least one clinical trait. The 19 DE miRNAs belong to 6 clusters identified in previous trajectory analyses are provided. AFC: antral follicle count; AG: accelerated growth; AMH: anti-Müllerian hormone; AR: accelerated recession; CR: convex recession; DG: decelerated growth; E2: estradiol; ET: endometrial thickness; LG: linear growth; LR: linear recession; P: progesterone.

^a^hsa-miR-34c-5p is negatively correlated with ET, and hsa-miR-6741-3p is positively correlated with ET.

### Target gene prediction of DE miRNAs

**Figure 4.**
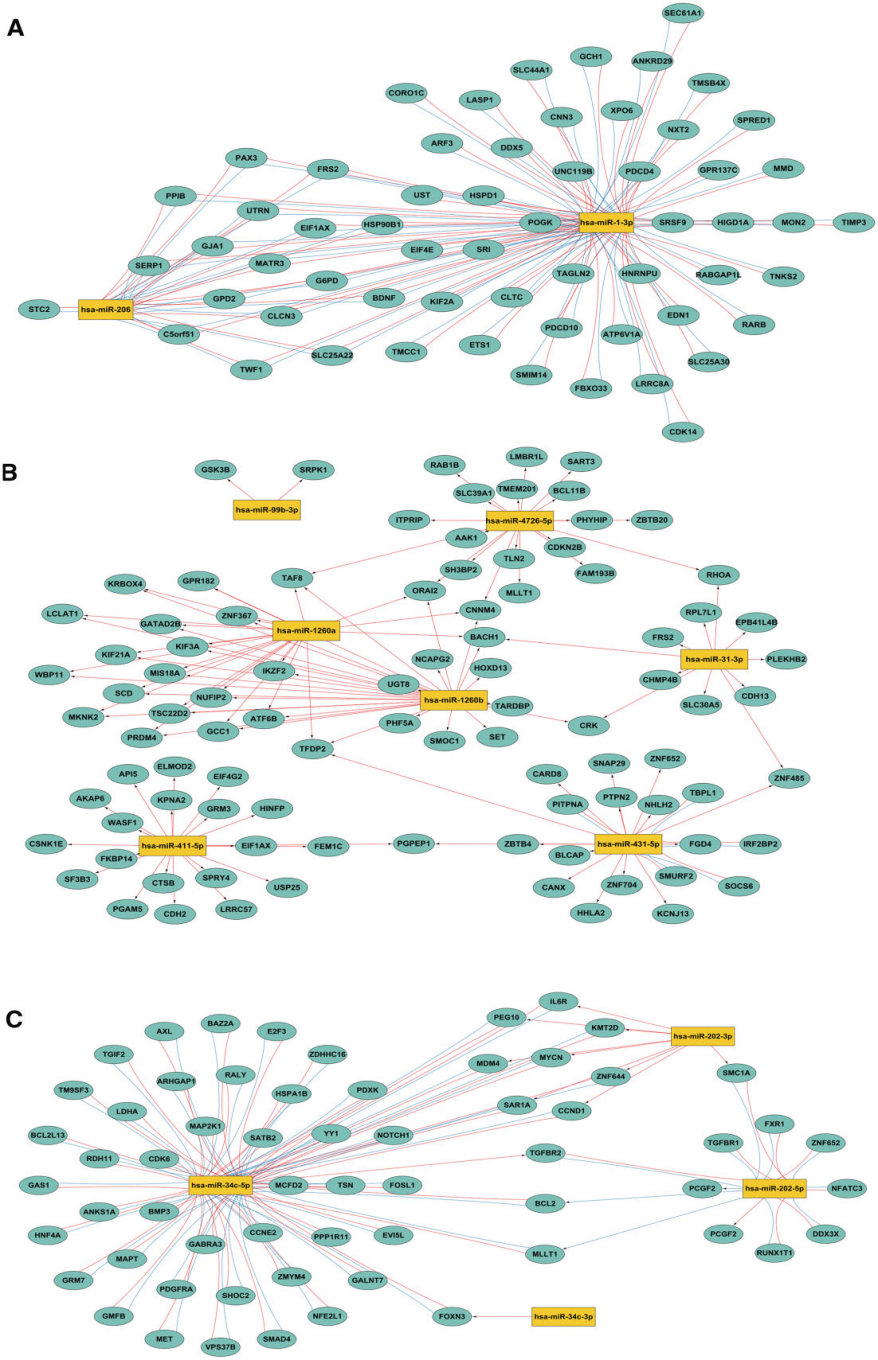
**Regulatory miRNA–mRNA networks.** Predicted targets for the clinically relevant differentially expressed miRNAs in pattern LR (**A**), LG (**B**), and AG (**C**). Owing to limited space, partial results are not displayed. The full list of predicted targets for miRNAs can be found in Supplementary Table S6. AG: accelerated growth; LG: linear growth; LR: linear recession.

To further explore the functions of those clinically related DE miRNAs, their gene targets were predicted by TargetScan, mirTarBase, and miRDB databases based on their expression pattern (Supplementary Table S6). For groups with more than one miRNA, the miRNA–target regulation network was constructed using Cytoscape (Fig. 4). In cluster LR (Fig. 4A), hsa-miR-1-3p and hsa-miR-206 were predicted as having multiple identical target genes, such as SERP1, HSP90B1, and G6PD. In cluster LG (Fig. 4B), five genes (BACH1, TAF8, TFDP2, CNNM4, and ORAI2) were simultaneously predicted as potential targets of three DE miRNAs. In cluster AG (Fig. 4C), IL6R was the common target of three miRNAs (miR-34c-5p, miR-6741-3p, and miR-202-3p). Notably, there were common genes targeted by miRNAs in the same cluster, indicating that those miRNAs showing similar expression patterns were functionally relevant.

**Figure 5.**
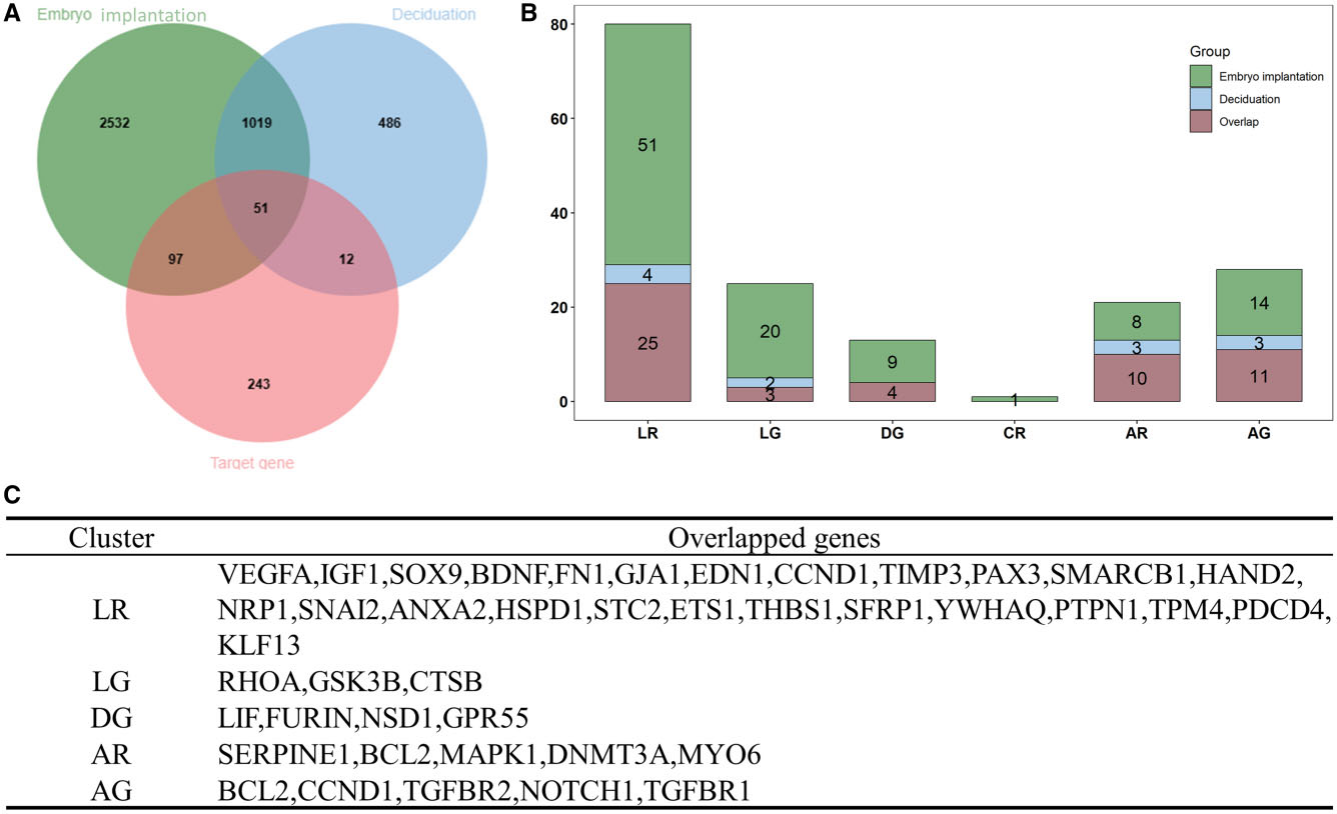
**Co-occurrence analysis results.** (**A**) Venn plot showing genes shared by embryo implantation-related genes, decidualization-related genes, and overlapped genes, identified through the target genes of the clinically related differentially expressed miRNAs. (**B**) Stacked bar chart shows the distribution of embryo implantation-related genes, decidualization-related genes, and overlapped genes in different clusters. (**C**) Overlapped genes in different clusters. AG: accelerated growth; AR: accelerated recession; DG: decelerated growth; LG: linear growth; LR: linear recession.

Furthermore, we investigated whether the 403 prediction targets of 19 DE miRNAs were associated with endometrium decidualization and embryo implantation. The information for genes linked with decidualization and embryo implantation (Supplementary Tables S7 and S8) was downloaded from GeneCards database (https://www.genecards.org/) by searching key words (decidualization or embryo implantation). As presented in Fig. 5A, there were 160 (39.7%) predicted genes matching gene sets involved in decidualization or embryo implantation, while 51 predicted targets were involved in both sets. Figure 5B shows the distribution of decidualization-related genes, embryo implantation-related genes, and overlapped genes in six different expression patterns. The data show that cluster LR contained the largest set of embryo implantation-related genes and overlapped genes (n = 25), and the number of overlapped genes in cluster AR and AG was 10 and 11, respectively. Additionally, the overlapped genes in different miRNA expression patterns were distinct (Fig. 5C), suggesting that DE miRNAs may interact with different target molecules during the WOI.

### Enrichment analysis of the predicted target genes

**Figure 6.**
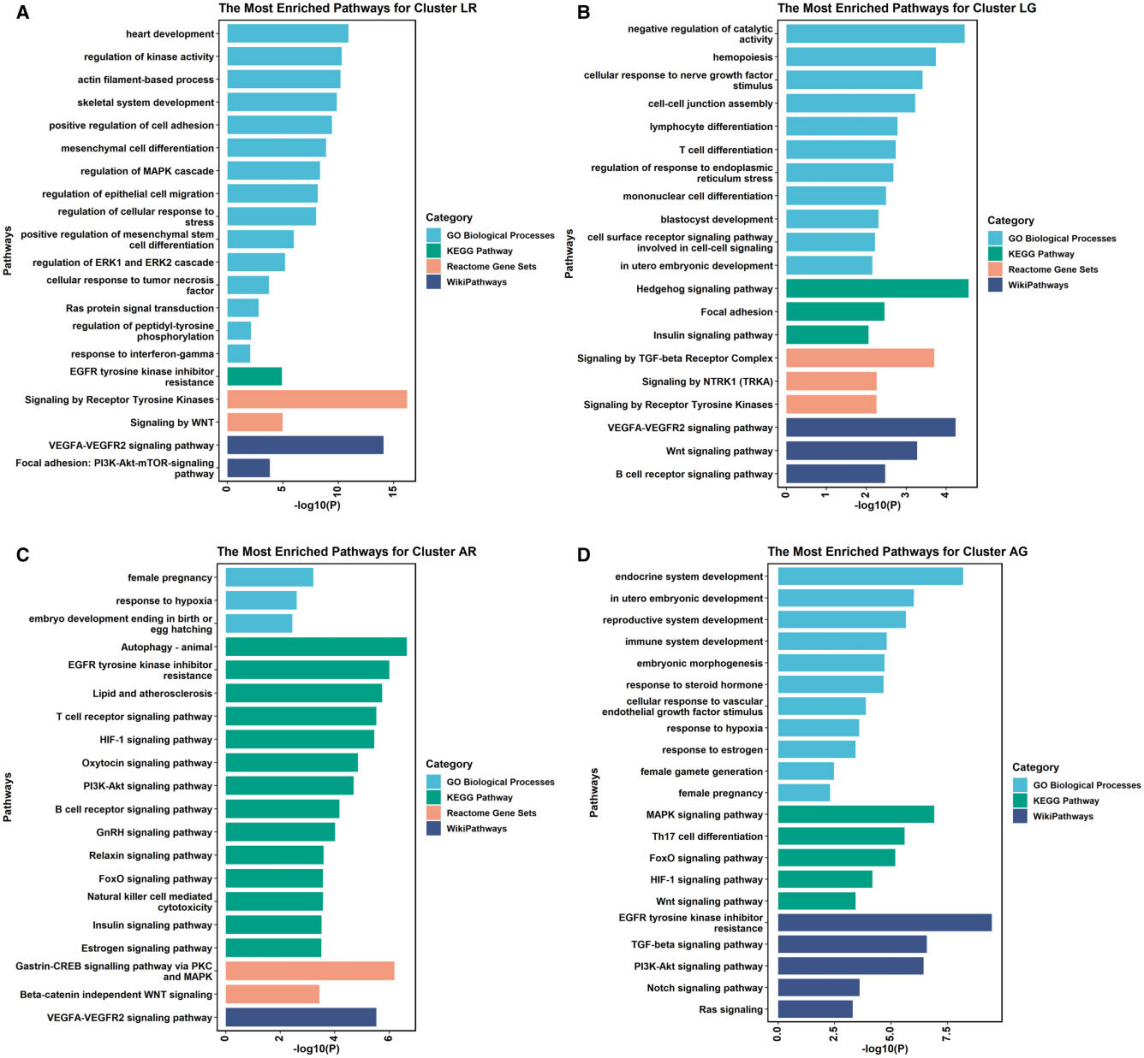
**Functional enriched signaling pathway.** Gene Ontology (GO) biological process, Kyoto Encyclopedia of Genes and Genomes (KEGG) Pathway, Reactome Gene Sets, and WikiPathway terms are searched and enriched by Metascape. The most enriched pathways for the predicted targets of differentially expressed miRNAs in cluster LR (**A**), cluster LG (**B**), cluster AR (**C**), and cluster AG (**D**). All bars represent log_10_-transformed adjusted *P*-values. AG: accelerated growth; AR: accelerated recession; LG: linear growth; LR: linear recession.

Having identified the predicted targets of 19 clinically related DE miRNAs, we further carried out an enrichment analysis to help understand their biological functions underlying the physiological process of embryo implantation. Functional annotation was conducted within each dynamic changing cluster. The most enriched biological pathways for cluster LR, LG, AR, and AG are displayed in Fig. 6. Interestingly, the vascular endothelial growth factor (VEGF) and Wnt signaling pathways were enriched in all four clusters, demonstrating the vital role of the two pathways during WOI as well as for embryo implantation. Additionally, both LR and LG clusters showed enriched functions related to cell adhesion or the cell–cell junction (Fig. 6A and B), which is essential for embryo implantation and establishment of pregnancy. Furthermore, the enrichment pathways concerning the target genes of DE miRNAs in cluster LG included ‘in utero embryonic development’, ‘insulin signaling pathway’, and ‘TGF-beta signaling’ (Fig. 6B). As presented in Fig. 6C, multiple biological processes or signaling pathways important for embryo implantation were enriched, such as ‘female pregnancy’, ‘response to hypoxia’, and ‘autophagy’, as well as HIF-1, PI3K-AKT, FoxO, insulin, and estrogen signaling pathways. Similarly, for cluster AG (Fig. 6D), some significant biological activities and pathways were also enriched, including ‘*in utero* embryonic development’, ‘response to steroid hormone’ and ‘response to hypoxia’, and ‘female pregnancy’, as well as FoxO, HIF-1, TGF-beta, PI3K-AKT, NOTCH, and Ras signaling pathways. The functional annotations performed for clusters DG and CR are shown in Supplementary Fig. S7. hsa-miR-4286 in the DG pattern was positively associated with P levels and was enriched in inflammatory responses and MAPK signaling pathways (Supplementary Fig. S7A), supporting its role in the establishment of maternal immune tolerance to the fetus through progesterone and the MAPK signaling pathway. However, biological pathways enriched for cluster CR were limited (Supplementary Fig. S7B), indicating that its biological functions may need further investigation. Nevertheless, according to the enrichment results, it is supposed that DE miRNAs distributed in different clusters may participate collectively in early pregnancy establishment.

### Assessment of miRNA expression in the validation set

To assess the robustness of all identified DE miRNAs, we further performed association tests in a validation set. The validation set was collected and included 115 samples (23 individuals with five time points) with successful pregnancy and 95 samples (19 individuals with five time points) with no successful pregnancy. The baseline characteristics are presented in Supplementary Table S9. All 46 DE miRNAs were further examined for associations between any two time points. DE miRNAs with the same effect directions as presented in the screening set, which are significant in the women with successful pregnancy in the validation set but not in those with an unsuccessful pregnancy, were deemed as replicative DE miRNAs. A total of four miRNAs (hsa-miR-202-3p, hsa-miR-328-3p, hsa-miR-6781-5p, and hsa-miR-1249-3p) were replicative DE miRNAs (Supplementary Fig. S8). Particularly, the differential expression between D0 and D7 was replicated for hsa-miR-202-3p, as well as D0 versus D3 and D0 versus D5 for hsa-miR-328-3p, D3 versus D9 for hsa-miR-6781-5p, and D3 versus D7 for hsa-miR-1249-3p. Additionally, we found that the four DE miRNAs had significantly different expression levels between successful and unsuccessful pregnancies at different time points. The global level of hsa-miR-202-3p (Supplementary Fig. S8A) was significantly lower in successful pregnancies than that in unsuccessful pregnancies while hsa-miR-6781-5p (Supplementary Fig. S8C) and hsa-miR-1249-3p (Supplementary Fig. S8D) had robustly higher levels in successful than unsuccessful pregnancies, and hsa-miR-328-3p did not show remarkable differences between the two groups (Supplementary Fig. S8B). The results may suggest that hsa-miR-202-3p, hsa-miR-6781-5p, and hsa-miR-1249-3p may be associated with embryo implantation or endometrium receptivity.

## Discussion

The endometrium undergoes a series of dynamic changes during the menstrual cycle until endometrial cells and glands achieve an optimum secretory state, providing a suitable uterine microenvironment for embryo implantation (Cha *et al.*, 2012). However, in women, implantation failure is a common event even if the maternal conditions are normal and the transferred embryos are of good quality. The incidence of pregnancy loss after embryo implantation is high, at ∼25–40% (Wilcox *et al.*, 1988), and 75% of failed human pregnancies are thought to result from implantation deficiency (Norwitz *et al.*, 2001). Many studies have revealed a strong association between endometrial (or uterine fluids) miRNAs and embryo implantation (Altmäe *et al.*, 2013, 2017; Choi *et al.*, 2016; Drissennek *et al.*, 2022; Ibañez-Perez *et al.*, 2022), but it was difficult to demonstrate regulatory roles for miRNAs in embryo implantation during the WOI because tissue sampling and embryo implantation are not performed in the same ovulation cycle. In contrast, in this study, by analyzing human plasma miRNA profiles during the entire WOI (at five different time points after ovulation) in women who received FET treatment, we first revealed the dynamic plasma miRNA expression features throughout the WOI and showed the potential relation between maternal circulating miRNAs and embryo implantation.

According to global statistical testing, 26 DE miRNAs were identified from D0 to D9, which exhibited an overall upregulation during the peri-implantation stage. According to the differential speed in growth, we classified the 26 DE miRNAs in three subgroups, including LG (n = 19), AG (n = 6), and DG (n = 1). Interestingly, the inflection points of AG and DG appeared nearby on the day of blastocyst transfer, meaning that certain miRNAs in the blood may undergo fluctuation around the day of blastocyst transfer. Moreover, by pairwise comparisons, we revealed additional 20 DE miRNAs between at least two time points of sampling. Similarly, those 20 miRNAs can be grouped into six expression patterns, consisting of AR, LR, CR, LG, AG, and CG. We found that AR, CR, AG, and CG groups, with variable expression levels along the time stage, showed a turning point around D5. The evidence shows that once P levels reach a critical threshold, a well-timed and orderly secretory transformation of endometrium begins (Franasiak *et al.*, 2016). The significant change of miRNA expression around D5 may indicate a rapid response to the event of embryo transfer, or the increasing P level during the peri-implantation stage, which may trigger changes in plasma miRNA expression.

To further understand the role of the DE miRNAs during the WOI, associations between expression of DE miRNAs and clinical traits were analyzed. Five miRNAs (miR-369-5p, miR-1260a, miR-1260b, miR-4286, and miR-411-5p) showed a negative correlation with basal LH level, but no relevant literature was found reporting a relation between those miRNAs and LH. Interestingly, we found that miR-202-3p had a strong positive correlation with AMH and AFC. Studies reported that miR-202 was predominantly expressed in the gonads in vertebrates and miR-202-3p may have a relation with oocyte quality or follicular development (Xie *et al.*, 2016; Gay *et al.*, 2018). However, the specific role of miR-202-3p in endometrium or embryo implantation during the WOI is not clear, although we found that the level of miR-202-3p was lower in successful pregnancies compared with unsuccessful pregnancies. Moreover, our data showed that miR-1-3p and miR-6741-3p were associated with endometrial thickness and P level during the WOI. One recent study demonstrated that miR-1-3p is expressed in endometrial tissue and/or fluids and is predicted to target P receptors (Nothnick, 2022); but at present, there are no studies reporting the role of miR-6741-3p in endometrium and in relation to P. miR-206, located on human chromosome 6p12.2, is highly conserved in genomic organization and sequence (Townley-Tilson *et al.*, 2010). As demonstrated in the MCF-7 breast cancer cell line, miR-206 can regulate an estrogenic response through EGFR signaling (Adams *et al.*, 2009), as well as inhibit IGF-1 protein expression, and low expression of miR-206 might contribute to endometrium angiogenesis during embryo implantation as evidenced in porcine endometrium (Hong *et al.*, 2019). Our data showed that the level of hsa-miR-206 was decreased from D0 to D9 and was negatively associated with dynamic P levels; we speculated that hsa-miR-206 may be affected by the gradual increase of P during the WOI. Additionally, we found that hsa-miR-31-3p and hsa-miR-34c-5p showed a significant negative correlation with ET. Some studies have reported that miR-34c-5p and miR-31-3p may be associated with endometriosis (Luo *et al.*, 2020; Szubert *et al.*, 2022), but whether the two miRNAs can directly regulate endometrium growth remains unclear. Collectively, although those DE miRNAs were shown to correlate with certain clinical traits, their specific functions in reproductive physiology need to be further investigated.

Generally, miRNAs regulate gene expression by binding to mRNAs in the cell cytoplasm. Instead of being translated into proteins, the marked mRNAs will be either degraded and their components recycled, or they will be preserved and translated later (Bartel, 2004). It should be noted that a single miRNA could have many target mRNAs owing to its short sequence, which is able to combine with different gene transcripts. To accurately find target genes, we predicted the potential target genes of the DE miRNAs from the different cluster groups by searching in the three different miRNA databases (TargetScan, mirTarBase, and miRDB). A total of 403 genes targeted by 19 DE miRNAs were predicted, and multiple genes associated with pregnancy establishment were uncovered, including SERP1, HSP90B1, G6PD, and IL-6R. Additionally, to further understand whether the predicted genes are associated with embryo implantation or decidualization, we performed a co-occurrence analysis in the GeneCards database, which recorded the genes related to the two biological events; we found that 51 genes predicted by the 19 DE miRNAs were involved in both embryo implantation and decidualization. We also observed that the overlapped genes in different miRNA clusters were varied, suggesting that DE miRNAs may combine with different target molecules involved in the process of embryo implantation or endometrium receptivity during the WOI.

Enrichment analysis for the predicted target genes of the DE miRNAs is helpful to further recognize the regulatory effects of those miRNAs during the WOI. According to our data, VEGF and Wnt signaling were enriched in four clusters. As an angiogenic factor, VEGF is capable of recruiting immune cells, regulating endometrial–epithelial cell adhesion, and stimulating angiogenesis at the implantation site, so that it is involved in embryo implantation by promoting embryo development, improving endometrial receptivity, and coordinating the communication between the embryo and endometrium (Guo *et al.*, 2021). Wnt signaling was shown to be a prerequisite for embryo implantation (Mohamed *et al.*, 2005). In our study, the miRNA target prediction showed that 10 miRNAs (hsa-miR-1-3p, miR-206, miR-411-5p, miR-4726-5p, miR-99b-3p, miR-31-3p, miR-1260b, miR-143-3p, miR-34c-5p and miR-202-5p) may be involved in VEGF and Wnt signaling. Additionally, other important biological processes and signaling pathways were enriched, such as ‘*in utero* embryonic development’, ‘insulin signaling pathway’, ‘TGF-beta signaling’, and ‘response to hypoxia’, as well as FoxO, HIF-1, TGF-beta, PI3K-AKT, NOTCH, and Ras signaling pathways. Those signaling pathways are believed to be necessary for embryo development and implantation (Ni and Li, 2017; Luo *et al.*, 2020; Sekulovski *et al.*, 2021). The enrichment results suggest that plasma miRNAs detected during the WOI may be engaged in the process of embryo implantation by coordinating some key genes linked with decidualization and implantation.

There are some limitations in this study. First, the characteristics of the circulating miRNA expression profiles in peripheral blood during the WOI may not fully explain the local molecular events surrounding embryo implantation. However, we compared the plasma miRNA expression patterns with endometrium tissue as well as uterine fluid and verified that miRNA expression patterns in plasma were similar to those of endometrium tissue collected at the secretory phase, suggesting that circulating miRNAs may have a close connection with the functional status of endometrial cells. Additionally, as the main aim of this study was to reveal the global dynamic changes in miRNA expression profiles before and after embryo implantation, we primarily analyzed the plasma DE miRNAs during the WOI among women undergoing embryo transfer, with a subsequent successful pregnancy. Although we also identified the DE miRNAs among successful and unsuccessful pregnancies using a validation cohort, we found that only four DE miRNAs differed significantly, and had the same effect directions, in successful pregnancies versus unsuccessful pregnancies. The differences in results between the screening and validation sets may be caused by the sample limitations. Future studies with larger cohorts are needed to further define differences in the plasma DE miRNAs in women with successful and unsuccessful pregnancies.

In summary, this study has revealed the dynamic changes in plasma miRNA expression profiles during the WOI when successful embryo implantation occurs. The constant changes in miRNA expression patterns in peripheral blood may reflect the progress of blastocyst embedding. Specifically, several miRNAs (such as hsa-miR-1-3p, miR-206, and miR-6741-3p) were predicted to be involved in the regulation of important molecular events in female pregnancy, including decidualization and embryonic development, by acting on the potential target genes or signal transduction pathways (such as VEGF, Wnt, TGF-β, HIF-1, and NOTCH). Overall, our study demonstrates the dynamic expression features of circulating miRNAs before and after embryo implantation during the WOI, and these findings may be helpful in understanding the physiological changes during early pregnancy in women with embryo implantation.

## Supplementary Material

hoad034_Supplementary_Data_File_S1

hoad034_Supplementary_Figures

hoad034_Supplementary_Tables

## Data Availability

The original data of small RNA sequence generated in this study have been uploaded to the Genome Sequence Archive (https://ngdc.cncb.ac.cn/gsa-human, GSA-Human: HRA005227). Other data underlying this article will be shared on reasonable request to the corresponding author.
