# Supplementary material for: Dynamic peripheral blood microRNA expression landscape during the peri-implantation stage in women with successful pregnancy achieved by single frozen-thawed blastocyst transfer
Source: Hum Reprod Open. 2023 Aug 29;2023(4):hoad034. doi: 10.1093/hropen/hoad034 (PMC10493182; doi:10.1093/hropen/hoad034)
Supplement: hoad034_Supplementary_Data_File_S1 [file hoad034_supplementary_data_file_s1.docx]

**Supplementary Data File S1**

**Blood Sample Collection and Processing**

Fresh peripheral blood samples were collected in EDTA tubes at the five time points (D0, D3, D5, D7 and D9). The day of ovulation assessed by gynecologist based on luteinizing hormone level and dominant follicle discharging was denoted as Day 0 (D0). The single blastocyst was transferred on D5. Briefly, a 5-mL peripheral blood sample were centrifuged at 2000×g for 10 minutes at 4℃, and supernatants were obtained, subsequently aliquoted, labelled and stored at −80℃ until analysis. Plasma samples were examined for hemolysis based on a two-step method. First, absorbance was measured at 414 nm with the use of a spectrophotometer (Thermo Scientific Nanodrop One) and samples with results ≤ 0.2 were considered as hemolysis-free samples which would receive small RNA sequencing. For samples with results＞0.2, delta quantification cycle (Cq) values for miR-23a miR-451 were further calculated. Samples with delta Cq <7 for these two miRNAs were selected as hemolysis-free samples for further analyses.

**Small RNA Sequencing Data analysis**

Raw reads were quality checked with the FASTQC software (version 0.11.9). Adapters were removed as required using the cutadapt software (version 3.4) (Martin, 2011) and tolerating 10% error. Reads without adapters or shorter than 18 bases, as well as reads with more than 3 N bases were discarded. The pre-processed reads were used as clean data (reads) for the following analysis. The samples with a lower than 20M bases or a lower than 90% Q30 of clean data were excluded.

To remove the non-miRNA sRNAs in clean reads as much as possible, we mapped the clean reads to a customized reference file using bowtie software (version 1.3.0) (Langmead, 2010). The following options were applied for the alignment process: -v1 (allowing one mismatch) -m50 (allowing up to 50 multi mapping events) – best –strata (only return one alignment with the best alignment score). The customized reference file included the sequence for tRNA, piRNA, rRNA, snRNA, snoRNA and other non-miRNA sRNAs (lncRNA, scRNA, etc.). Human tRNA reference were obtained from the GtRNAdb (<http://gtrnadb.ucsc.edu/genomes/eukaryota/Hsapi38/hg38-tRNAs.tar.gz>) (Chan and Lowe, 2016). Human piRNA reference were downloaded from the piRNABank (Sai Lakshmi and Agrawal, 2008). Human rRNA reference were downloaded from NCBI. The reference of snRNA, snoRNA and other sRNAs were obtained from Rfam database (version 14.5) (Nawrocki *et al.*, 2015). The unmapped reads with longer than 26 bases were removed, followed by collapsing reads to ensure that each sequence only occurs once. The collapsed filtered unmapped reads were used as the valid reads for the next step analysis.

**Batch effect detection**

The raw counts were transformed into log-form counts by assuming a Negative-Binomial (NB) distribution using the Variance Stabilizing Transformation (VST) method for unsupervised data visualization (Anders and Huber, 2010). Then, high-dimensional reduction techniques Principal Component Analysis (PCA) and *t*-SNE (*t*-distributed stochastic neighbor embedding) were employed using the PCA and Rtsne functions on the normalized data, respectively.

**Small RNA-Seq data processing and differential expression analysis**

To remove low count data, a row elimination was performed for zero-count in at least 5 replicates. For screening set, batch effect was further removed using ComBat_seq function in the R package sva. To study the dynamic changes in miRNA expression during peri-implantation period, tradeSeq was introduced to perform trajectory inference as well as identifying differentially expressed miRNAs. tradeSeq is a powerful generalized additive model framework based on the negative binomial distribution. Although it has been developed for application in single-cell transcriptome sequencing (scRNA-seq) data with pseudotime, the framework may also be applicable to bulk RNA-seq time-course studies (Van den Berge *et al.*, 2020). For each miRNA, we fit a general additive model (GAM) with parameter K (number of knots) of three to model the relationships between gene expression and time-point and tested for the significance of their associations using the associationTest function. We picked out the significant miRNA s with FDR-corrected *p* values <0.05 as dynamic differentially expressed (DE) miRNAs. For visualization, predictSmooth function was applied for the dynamic DE miRNAs to generate fitted values from the tradeSeq models. Then, pairwise comparison between two time points was performed using the startVsEndTest function. DE miRNAs were also identified based on significance level of adjusted p-values <0.05. For validation set, when the expression of all above DE miRNAs needed to be compared, it was normalized using VST method. Mann-Witney-Wilcoxon test was conducted for comparisons of miRNA expression between two time points. 0.05 significance level of *p*-values was used in above comparisons. The analysis was performed on R (V4.1.2). ComplexHeatmap (V2.8.0) (Gu *et al.*, 2016) and pheatmap (V1.0.12) were used for data visualization.

**References:**

Anders S, Huber W. Differential expression analysis for sequence count data. *Genome Biol.* 2010; **11**:R106.

Chan PP, Lowe TM. GtRNAdb 2.0: An expanded database of transfer RNA genes identified in complete and draft genomes. *Nucleic Acids Res*. 2016; **44**:D184–D189.

Gu Z, Eils R, Schlesner M. Complex heatmaps reveal patterns and correlations in multidimensional genomic data. *Bioinformatics*. 2016; **32**:2847–2849.

Langmead B. Aligning short sequencing reads with Bowtie. *Curr Protoc Bioinformatics*. 2010; Chapter 11:Unit-11.7. doi:10.1002/0471250953.bi1107s32

Martin M. Cutadapt removes adapter sequences from high-throughput sequencing reads. *EMBnet.journal*. 2011; **17**: 1–12.

Nawrocki EP, Burge SW, Bateman A, Daub J, Eberhardt RY, Eddy SR, et al. Rfam 12.0: Updates to the RNA families database. *Nucleic Acids Res*. 2015; **43**:D130–D137.

Sai Lakshmi S, Agrawal S. piRNABank: A web resource on classified and clustered Piwi-interacting RNAs. *Nucleic Acids Res*. 2008; **36**:D173–D177.

Van den Berge K, Roux de Bézieux H, Street K, Saelens W, Cannoodt R, Saeys Y, Dudoit, S, Clement L. Trajectory-based differential expression analysis for single-cell sequencing data. *Nat Commun.* 2020; **11**:1201.
