# Supplementary material for: Dynamic peripheral blood microRNA expression landscape during the peri-implantation stage in women with successful pregnancy achieved by single frozen-thawed blastocyst transfer
Source: Hum Reprod Open. 2023 Aug 29;2023(4):hoad034. doi: 10.1093/hropen/hoad034 (PMC10493182; doi:10.1093/hropen/hoad034)
Supplement: hoad034_Supplementary_Figures [file hoad034_supplementary_figures.docx]

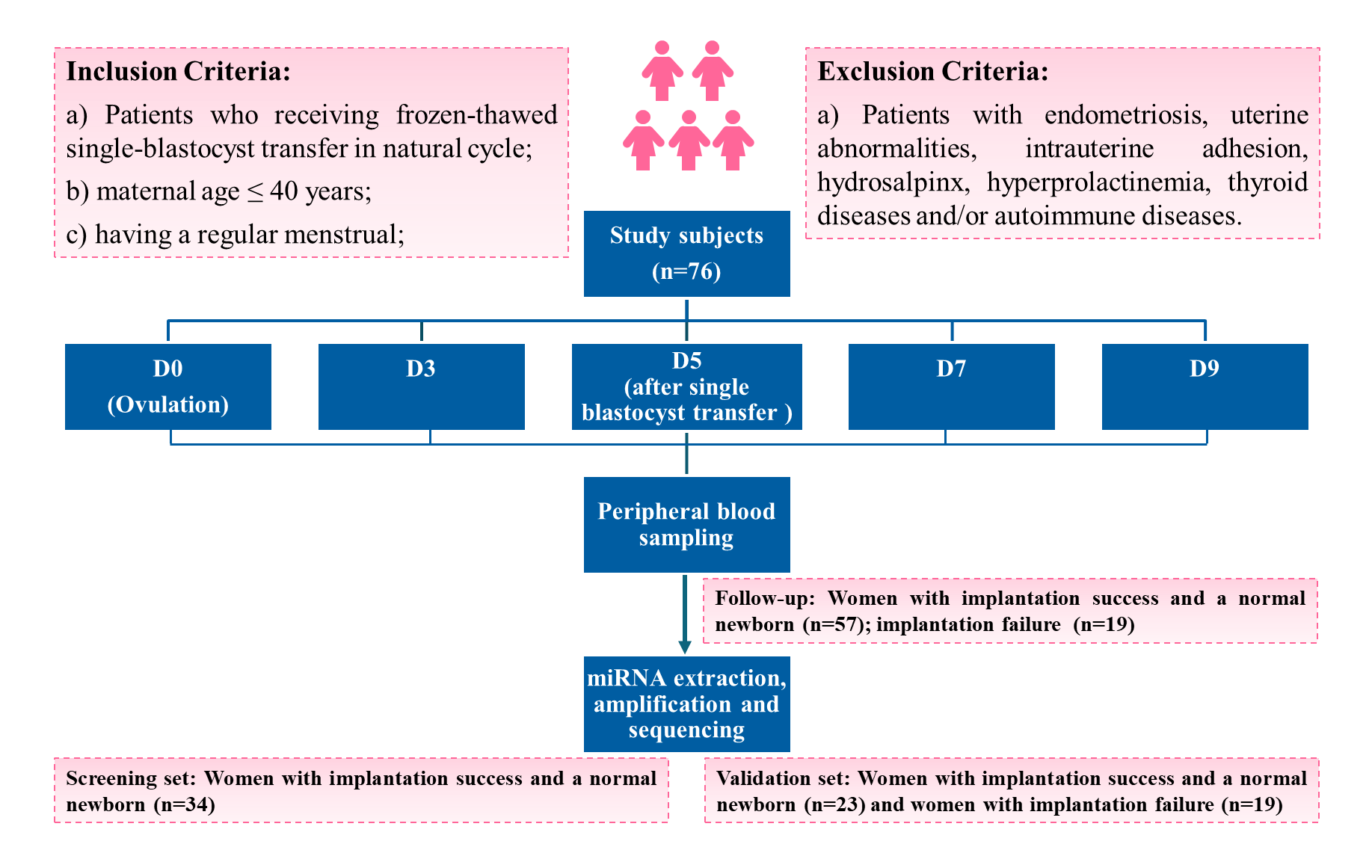


**Supplementary Figure S1** Overview of the study design.

The day of ovulation assessed by a gynecologist was based on plasma LH level and release of the dominant follicle was denoted as Day 0 (D0)


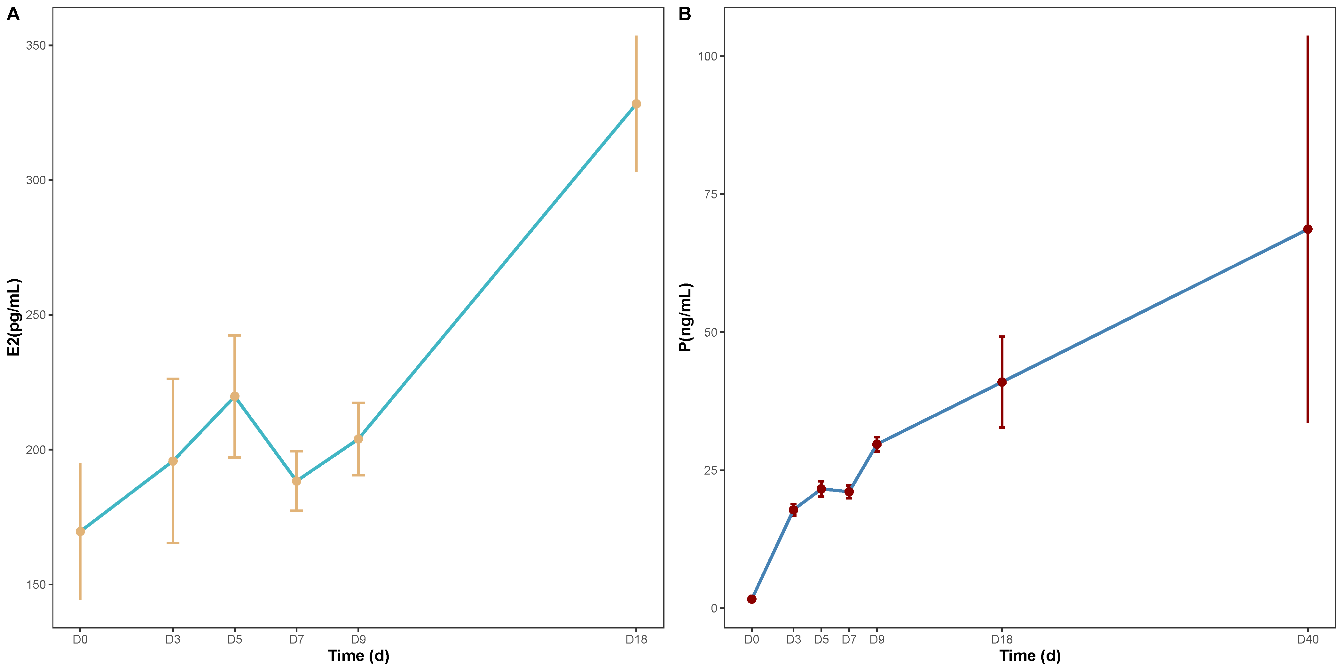


**Supplementary Figure S2** Line chart showing the dynamic changes in hormone concentrations during peri-implantation stage.

(A) estradiol (E2) and (B) progesterone (P). Time (d) starts from the day of ovulation, e.g. Day 0 (D0) refers to the ovulation day and D9 refers to 9 days after the ovulation day.


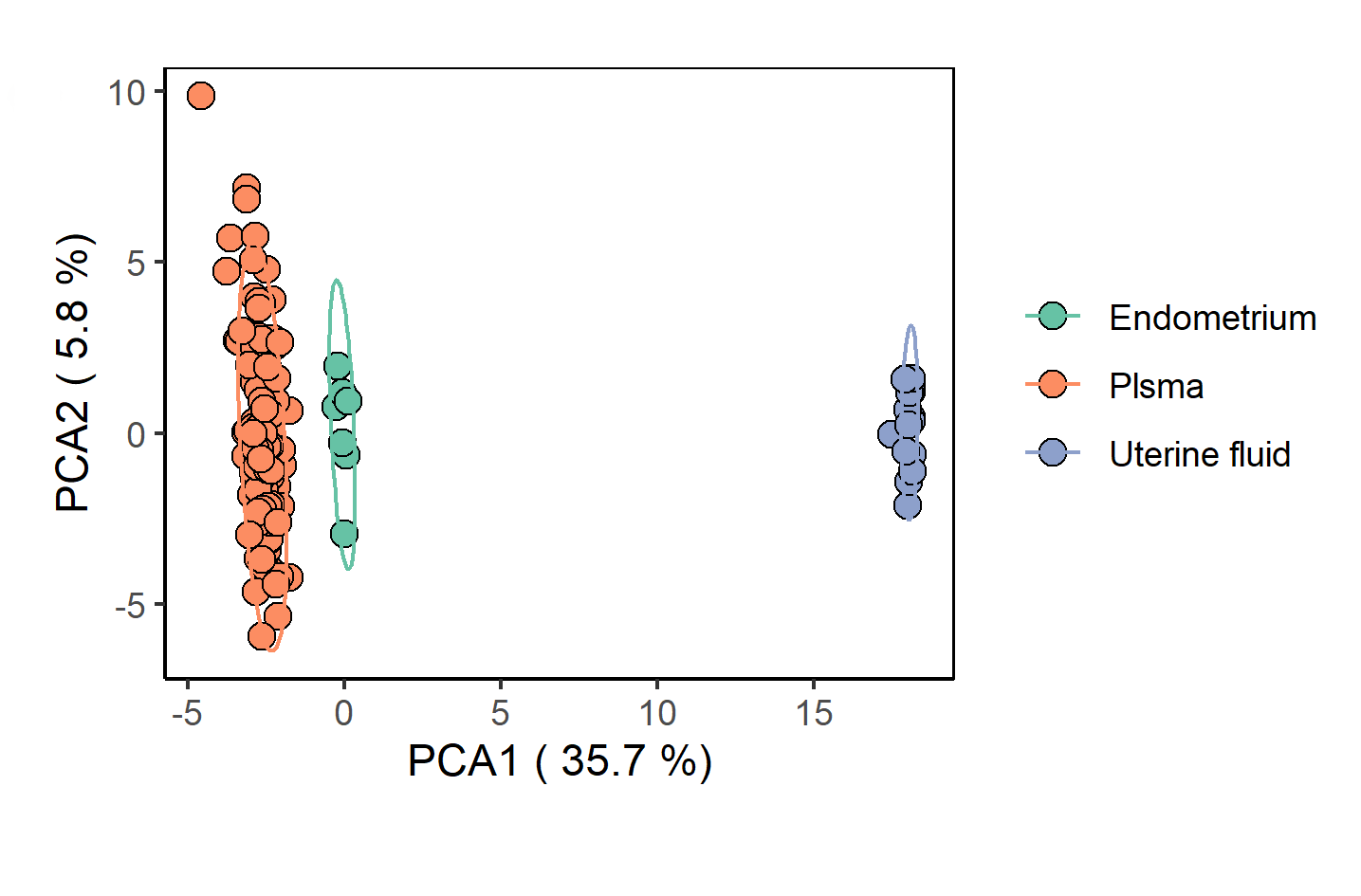
 **Supplementary Figure S3** Tissue similarity transcription analysis.

PCA of plasma miRNAs, comparing with the transcriptional profile of endometrium and uterine fluid miRNAs at the mid-secretory phase. Tissue-resident miRNAs are collected from published expression sets (GSE86491, GSE173289).


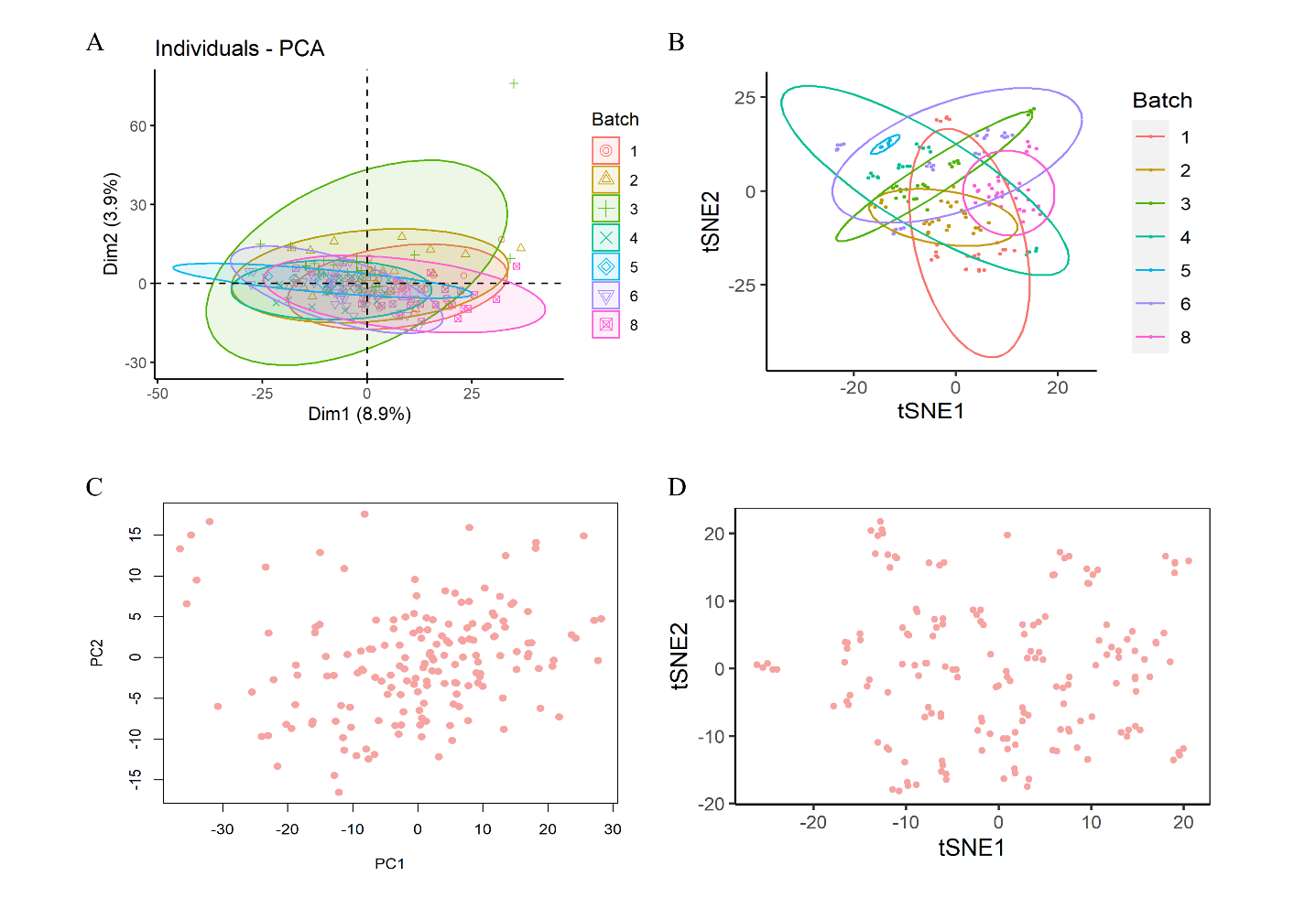


**Supplementary Figure S4** Principal Component Analyses and T-SNE of miRNA sequencing experiments for outlier detection.

(A) principal component analysis (PCA) and (B) T-SNE for the total miRNA profile for screening set.


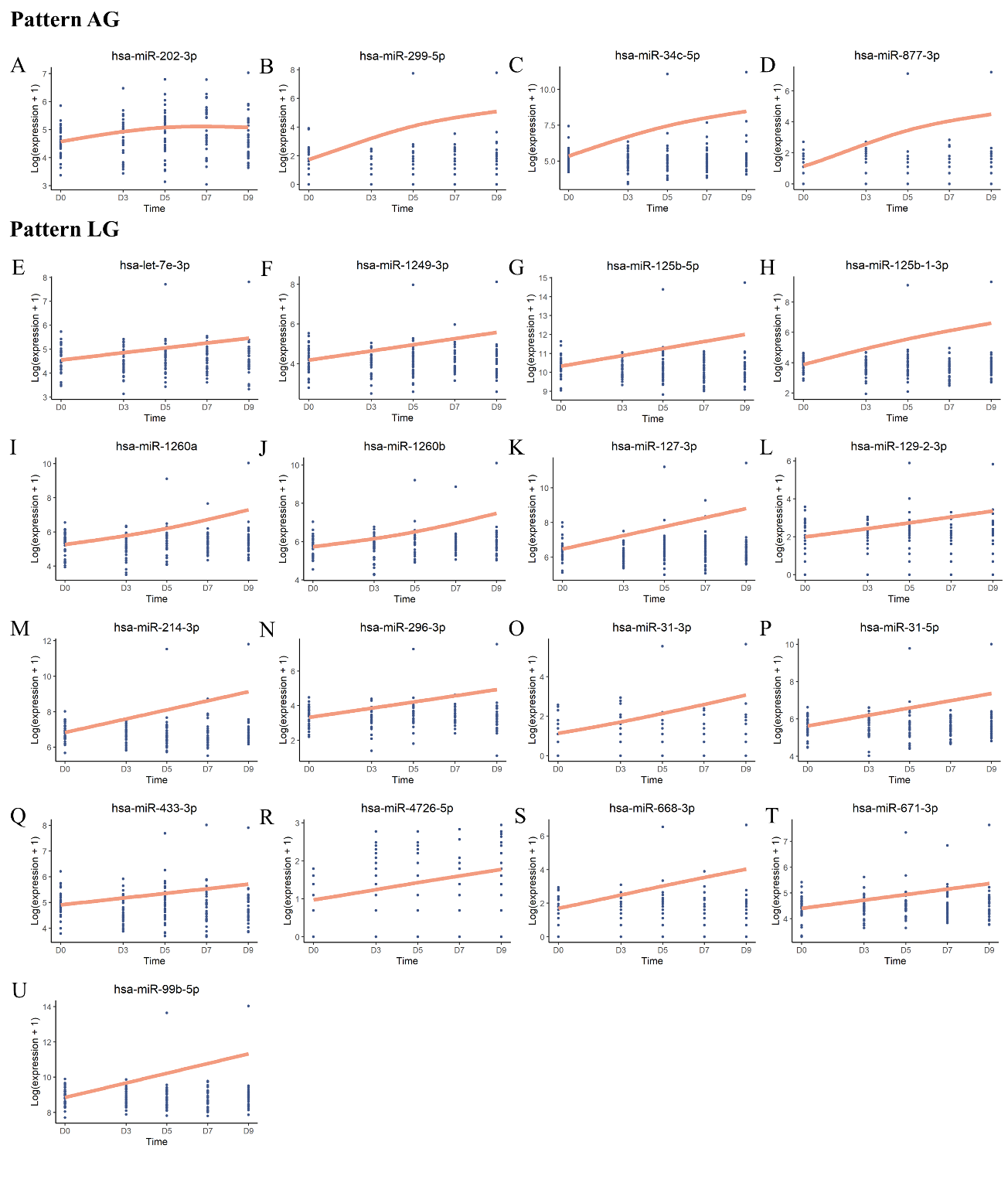


**Supplementary Figure S5** The visualization of the expression pattern of dynamic differential expression miRNAs.

(A-D) show the expression pattern of dynamic differentially expressed (DE) miRNAs in pattern AG. (E-U) show the expression pattern of dynamic DE miRNAs in pattern LG. AG: accelerated growth, LG: linear growth.


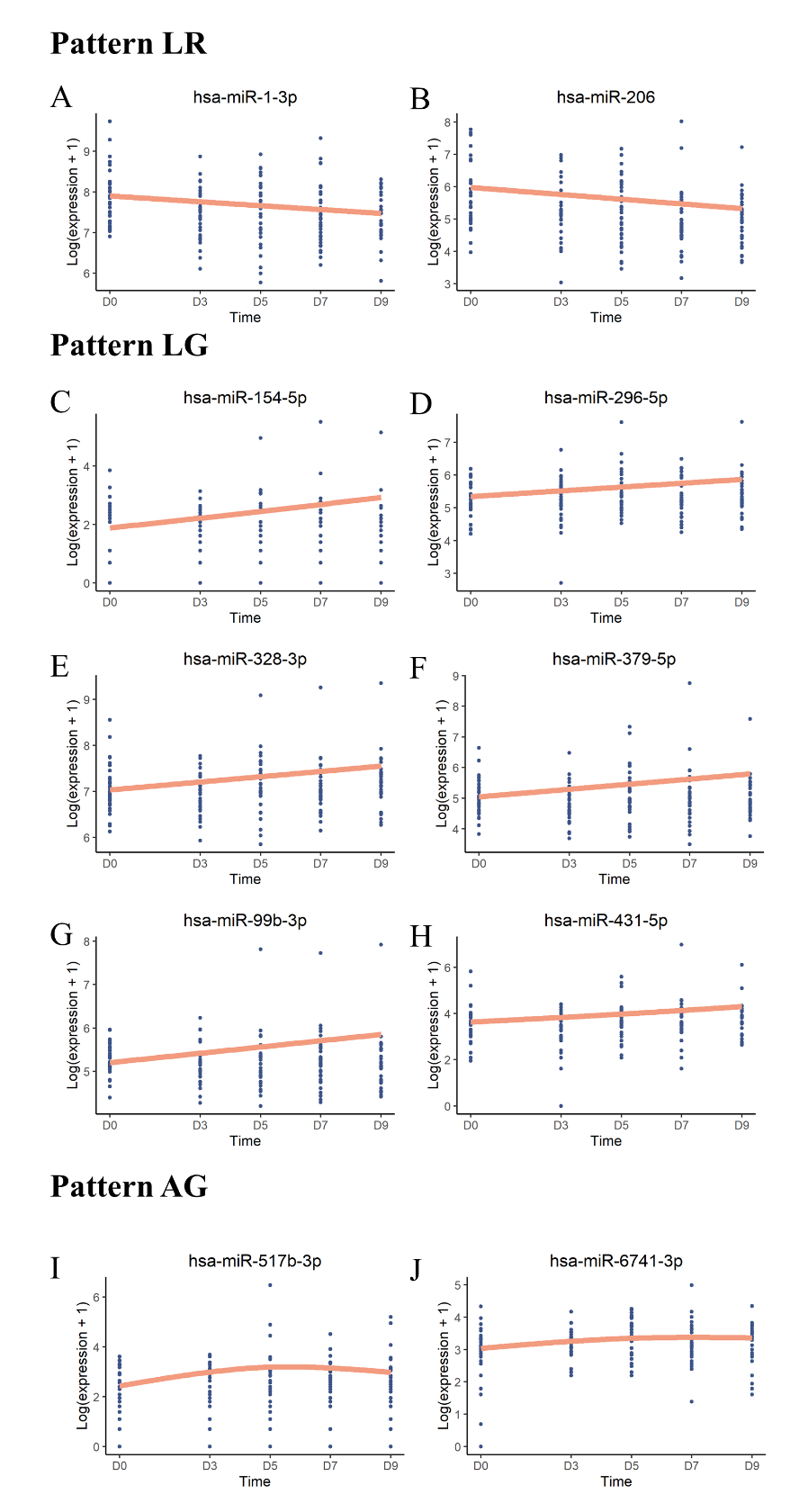


**Supplementary Figure S6** The visualization of the expression pattern of pairwise differential expression miRNAs.

(A-B) show the expression pattern of dynamic differentially expressed (DE) miRNAs in pattern LR. (C-H) show the expression pattern of dynamic DE miRNAs in pattern LG. (I-J) show the expression pattern of dynamic DE miRNAs in pattern AG. LR: linear recession, LG: linear growth, AG: accelerated growth.


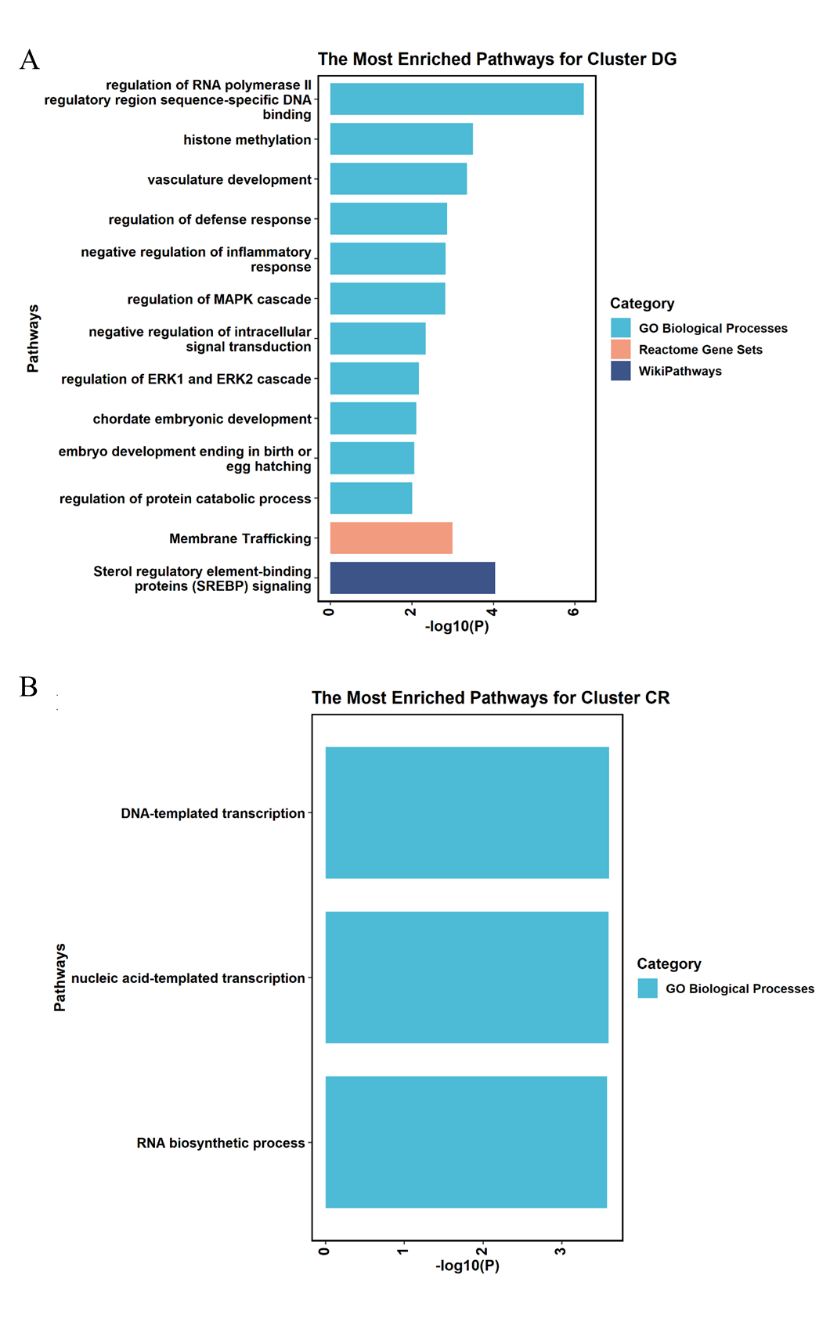


**Supplementary Figure S7** Functional enriched signaling pathway.

Gene Ontology (GO) biological process, Kyoto Encyclopedia of Genes and Genomes (KEGG) Pathway, Reactome Gene Sets and WikiPathway terms are searched and enriched by Metascape. (A-B) The most enriched pathways for the predicted targets of differentially expressed miRNAs in cluster DG (A) and cluster CR (B). All bars represent log_10_ transformed adjusted *p*-values. DG: decelerated growth, CR: convex recession.


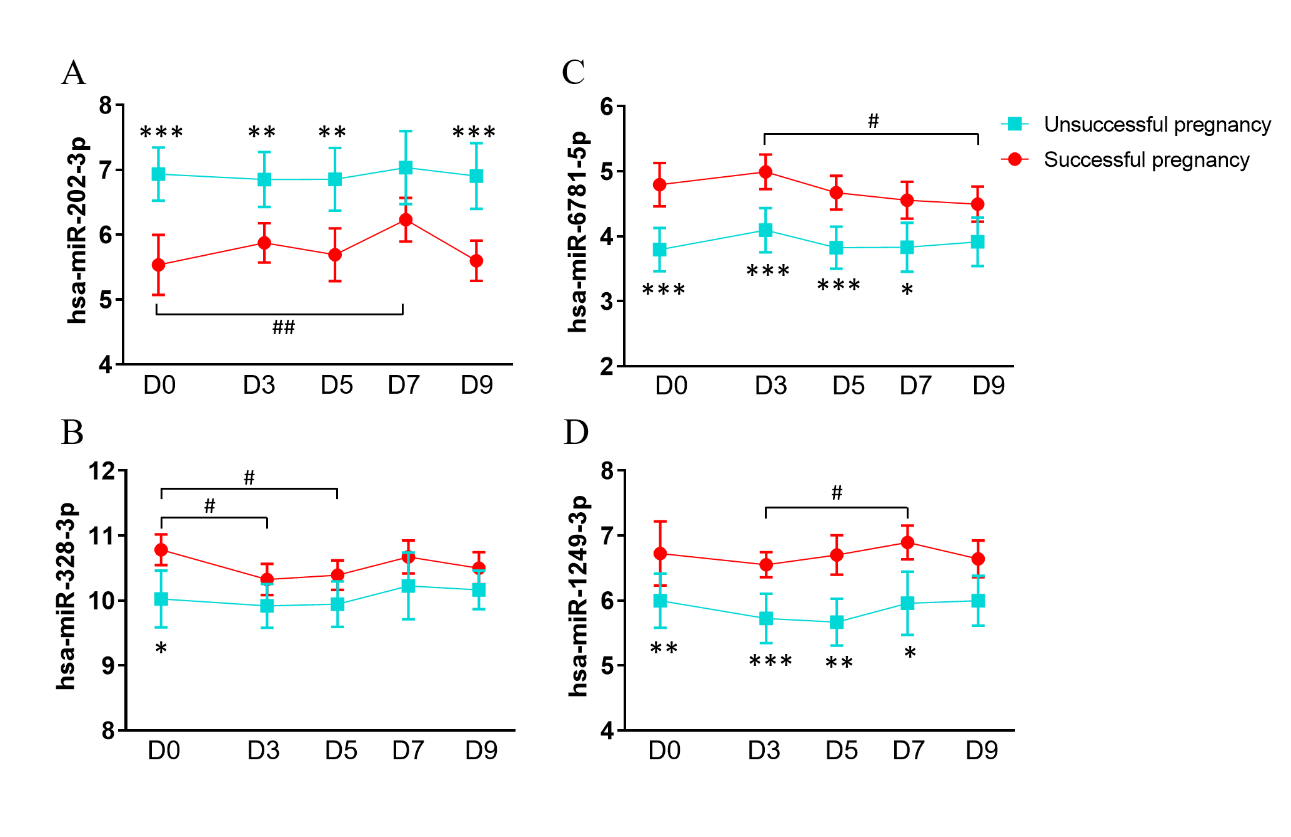


**Supplementary Figure S8** Assessment of miRNA expression in the validation set.

The validation set includes 23 individuals with successful pregnancy and 19 individuals with unsuccessful pregnancy. Four DE miRNAs are identified with significant change as shown in screening set. (A-D) show the expressions of DE miRNAs ((hsa-miR-202-3p, hsa-miR-328-3p, hsa-miR-6781-5p and hsa-miR-1249-3p) between successful and unsuccessful pregnancies. Two-way ANOVA was used for statistical analysis. #comparisons were between different time points in successful pregnancies (#*p-*value<0.05). *comparisons were performed between successful and unsuccessful pregnancies (**p-*value<0.05, ***p-*value<0.01, ****p-*value<0.0001).
